# Quality of life of older frail persons receiving a post-discharge program

**DOI:** 10.1186/1477-7525-11-58

**Published:** 2013-04-12

**Authors:** Tracy A Comans, Nancye M Peel, Leonard C Gray, Paul A Scuffham

**Affiliations:** 1 School of Medicine, Griffith University, Brisbane, Australia; 2 Population and Social Health Research Program, Griffith Health Institute, Brisbane, Australia; 3 Centre for Research in Geriatric Medicine, The University of Queensland, Brisbane, Australia

**Keywords:** Quality of life, Frail older, Economics, Community health services

## Abstract

**Background:**

A key goal for services treating older persons is improving Quality of Life (QoL). This study aimed to 1) determine the QoL and utility (i.e. satisfaction with own quality of life) for participants of a discharge program for older people following an extended hospital episode of care and 2) examine the impact of the intensity of this program on utility gains over time.

**Methods:**

A prospective observational cohort study with baseline and repeated measures follow up of 351 participants of the transition care program in six community sites in two states of Australia was conducted. All participants who gave consent to participate were eligible for the study. QoL and utility of the participants were measured at baseline, end of program, three and six months post baseline using the EQ-5D and ICECAP-O. Association between the intensity of the program, measured in hours of care given, and improvement in utility were tested using linear regression.

**Results:**

The ICECAP-O yielded consistently higher utility values than the EQ-5D at all time points. Baseline mean (sd) utility scores were 0.55 (0.20) and 0.75(0.16) and at six months were 0.60 (0.28) and 0.84 (0.25) for the EQ-5D and ICECAP-O respectively. The ICECAP-O showed a significant improvement over time. The intensity of the post-acute program measured by hours delivered was positively associated with utility gains in this cohort.

**Conclusions:**

A discharge program for older frail people following an extended hospital episode of care appears to maintain and generate improvements in QoL. The amount of gain was positively influenced by the intensity of the program.

## Background

The lack of residential care places or alternative community care contributes to a term known as a “bed blocker” – an older person who has a lengthy hospital stay and is unable to be readily discharged due to functional decline. This causes congestion throughout the hospital system with more acute patients unable to be admitted, lengthy delays in emergency departments and reduction in elective surgery output. These system pressures have facilitated the development of post-discharge programs with the expectation that they will reduce length of stay by allowing earlier discharge from hospital or by providing slow stream rehabilitation services in the community rather than in a sub-acute hospital setting [1].

A key goal for community services for older persons is improving Quality of Life (QoL) [2]. Previous research has indicated that older persons transitioning from hospital to residential aged care (RAC) have very poor health-related QoL [3], however, it is not clear how delaying entry to RAC and/or allowing a period of convalescence or slow stream rehabilitation at home might affect an older person’s QoL after the acute hospitalisation period.

The Australian Transition Care Program is a clearly defined post-acute discharge program for older people. To be eligible, an older adult must be assessed as needing the level of support of at least low level residential care while in hospital for an acute care episode. Services provided by the transition care program include case management, medical and nursing support, rehabilitation services and personal and domestic care over a maximum period of 12 weeks (average seven weeks) post discharge from hospital [4]. The aim of the transition care program is to enable older persons to return home, rather than prematurely enter residential care, and to optimise their functional capacity. Additional aims are to promote earlier discharge to free hospital beds for other uses and to minimise the chance of re-admission [5].

Utility is an important construct used in economic evaluation to measure the level of satisfaction with a person’s quality of life and is usually anchored between 0 (death) and 1 (representing full health). There is a lack of research on the effect of community service programs, such as transition care, on QOL, nor has the influence on QoL of program intensity, measured in the actual patient contact time spent delivering services, been examined. Additionally, while previous studies have reported overall summary measures of QoL, specific information on what domains are most influenced by particular programs for older people is lacking.

The aims of this study were to:

1. Determine the QoL and utility for participants of a post-acute hospital program.

2. Examine the impact of the intensity of a post-acute hospital program on utility gains over time.

## Methods

### Study design

A prospective observational cohort study with baseline and repeated measures was conducted in six community based transition care programs in two states of Australia, Queensland and South Australia. Data were collected at four time points: Admission to transition care program, discharge from transition care program and at three and six months post admission to the transition care program. Ethical approval for the study was granted by the University of Queensland Human Research Ethics Committee (HREC) (2009001647) as well as HRECs responsible for governance at each of the transition care program sites.

### Subjects

Included in the study were all consenting patients entering the community-based transition care program, immediately following a hospitalisation and/or temporary residential care stay. No exclusion criteria were applied. A detailed description of the participants has been provided previously [6].

### Assessments

Demographic details were collected via the interRAI Home Care tool [7]. This tool is one of a suite of assessment tools designed to support the assessment and care planning of older frail people; it provides a seamless assessment process across settings reducing duplication in assessment for the patient and health professionals [7]. Frailty was defined by calculating a frailty index from the accumulation of deficits in interRAI Home Care domains (including cognition, communication, mood, functional status, continence, health conditions and symptoms, nutritional status, and number of medications) using the method specified by Rockwood [8]. A higher frailty index indicates increasing frailty and a threshold of 0.25 has been proposed as the demarcation between ‘fitness’ and ‘frailty’ in community-dwelling older people [6,9].

Health related QoL was measured using the EQ-5D, a reliable and valid tool that has been extensively used in similar patient populations [10-12]. The EQ-5D measures health in five domains: mobility, self-care, usual activities, pain and anxiety/depression. The summary score (representing a person’s utility) derived from the EQ-5D is anchored at 0 (representing death) and 1.0 (representing full health). Scores below zero are possible on this scale representing states considered worse than death.

A measure of capability, the ICECAP-O, was also collected. The ICECAP-O measures wellbeing and quality of life in a wider sense and was designed from the theoretical basis of the capabilities framework derived by Sen [13]. This approach attempts to measure what the person is capable of performing rather than what health state the person is in. The tool measures five attributes: Attachment, Security, Role, Enjoyment and Control with four levels in each attribute ranging from best to worst. For example, the Security levels are:

1. I can think about the future without any concern

2. I can think about the future with only a little concern

3. I can only think about the future with some concern

4. I can only think about the future with a lot of concern

It has been designed to be converted to a utility measure for possible use in economic evaluations with a range of 0 (representing no capability) to 1.0 (representing full capability) [14].

Service characteristics were collected in conjunction with the cohort study by the service providers. These were the hours spent with each patient by various service providers (for example, total physiotherapy visits and time for an episode of care), travel time, case management time, wage rates of staff and equipment provided.

### Statistical analyses

Descriptive statistics are presented for the domain scores and the summary scores for both the EQ-5D and ICECAP-O. Summary scores for the EQ5D were calculated using the Australian algorithm [15] and for the ICECAP-O using the UK weighting system [16]. The change in utility over time was analysed using a generalized estimating equation (GEE) with a Gaussian family and exchangeable correlation structure. The GEE is a flexible way of analysing repeated measures within an individual and can accommodate missing data [17].

Quality adjusted life years (QALYs) reflect both the quality and quantity of time lived. For example, six months in full health would generate 0.5 QALYs. While QALYs are commonly calculated using EQ-5D utility values, the ICECAP-O has not necessarily been designed to capture this measure. For the purposes of comparison, a similar time × utility measure was calculated for this study. QALYs over the study period were calculated for the EQ-5D using the area under the curve of the four measurement points and an equivalent measure (utility × time) was generated for the ICECAP-O [18]. In order to measure whether a positive or negative change in utility had occurred from baseline, incremental QALYs (or utility × time) were calculated as the change from baseline area under the curve. A paired *t*-test was used to compare the resultant incremental change scores.

The service time (e.g. nursing, physiotherapy) is presented by mean (standard deviation) and by percentage of participants receiving that service.

The association between the QALYs gained from the EQ-5D or ICECAP-O (dependent variable) and the service time input of the various components (physiotherapy, nursing etc.) of the program (independent variables) were examined separately using univariate linear regression analyses. The significance level was set to 0.05. All analyses were performed using STATA 12®.

A small amount of missing data for the QoL questionnaires was present (<3%) either because the entire questionnaire was missing at that time point or because the questionnaire was incomplete. Examination of the data visually using scatterplots indicated that there were no patterns present and data appeared to be Missing Completely at Random. Given the small numbers and random nature of the “missingness”, it was considered that this would not impact on the overall results and therefore no imputation was necessary. Where data was present in an incomplete questionnaire it has been used in the presentation of the domain scores, however, the total utility score was unable to be calculated for these participants.

## Results

A total of 351 participants were recruited to the trial from November 2009 to September 2010. Follow-up was completed in April 2011 and demographic details are provided in Table 1.

**Table 1:** Descriptive characteristics of participants

**Characteristic**		**Sample size (N = 351)**
Age	Mean (SD)	78.99 (8.80)
Gender	Females	231 (66%)
Living arrangements	Lives alone	171 (49%)
	Lives with spouse	131 (37%)
	Lives with others	49 (14%)
Length of stay (days) in hospital prior to TCP	Median (IQR)	27 (16–45)
Length of stay (days) in TCP	Median (IQR)	54 (37–73)
No. of medications at TCP admission	≤ 3	22 (6%)
	4–5	32 (9%)
	6–8	91 (26%)
	9–12	123 (35%)
	13–16	71 (20%)
	≥ 17	8 (2%)
	Missing data	4 (1%)
No. of co-morbidities at TCP admission	Mean (SD)	6.02 (3.06)
Frailty Index	Mean (SD)	0.31 (0.10)

TCP = transition care program.

Participants were older people with an average age of 79 years, had multiple co-morbidities and polypharmacy and showed a mean Frailty Index (FI) of 0.31; indicating the program appropriately targeted a frail older population. Around 87% of participants remained at home at the six-month follow-up point.

At three months, 15 people (4.7%) had died and this increased to 22 (6.3%) at study completion. Of the 22 deaths, 10 were male (8% of all males) and 12 female (5% of all females). This corresponds to an annual mortality rate of 0.16 for males and 0.10 for females. Comparison with Australian life tables [19] for the average age of males of 78 (annual death rate = 0.044) and of females of 80 (annual death rate = 0.036) demonstrates that the risk of dying was around three times higher for participants of transition care than that of the general Australian population of the same age.

**Figure 1:**
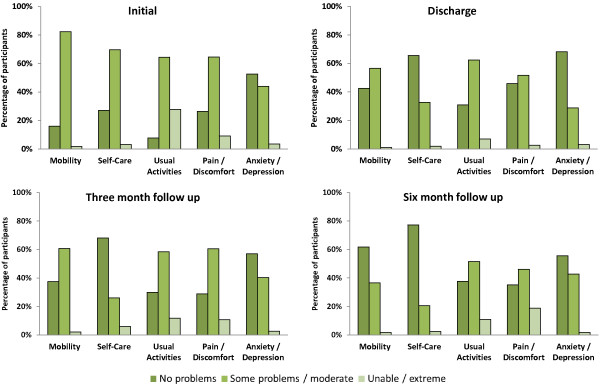
Change over time of the domains of the EQ-5D.

The results of the domains of the EQ-5D are given in Figure 1. The majority of participants had problems with mobility, self-care and usual activities at baseline. The results in these three domains show a rapid improvement from baseline to discharge from the transition care program. Assuming baseline values as reference, the improvement in mobility, self-care and usual activities was confirmed even at three and six month follow-up.

The profile of pain differed from the dimensions of mobility, self-care and usual activities presented above. While there does appear to be some improvement in the percentage of people reporting no pain and a reduction in those reporting some or extreme pain by the end of transition care, this improvement appears to have been reversed to baseline levels by three months. The profile of anxiety/depression is also broadly similar to that of pain. Interestingly, although the participants had high levels of functional and mobility problems, over half of participants reported no anxiety or depression at all four time points. Only a small percentage of people (3.4%) rated themselves as extremely anxious or depressed on admission to transition care and this percentage was similar at the end of the episode of care (3.1%).

**Figure 2:**
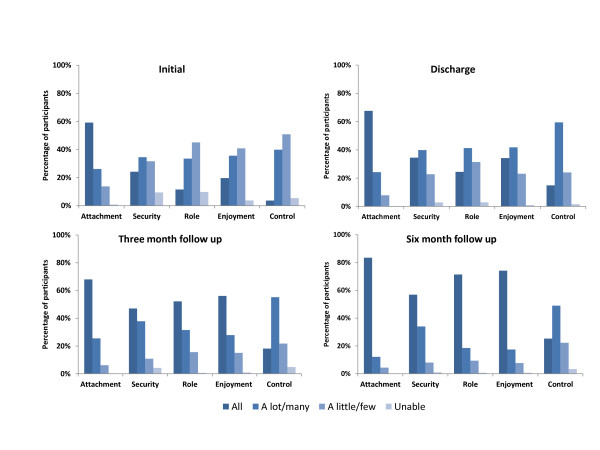
**Change over time of the domains of the ICECAP-O.** Note: Please refer to the ICECAP-O scale for clarification of the levels displayed [14].

For the ICECAP-O the attributes that participants reported being the most limited at baseline were control (being independent) and role (doing things that make you feel valued) (Figure 2). Only 11% of people rated themselves as able to do all the things that made them feel valued initially; this proportion had doubled by the end of the transition care program episode and had risen to 50% by three months. All attributes apart from attachment showed improvement at all three follow up time points indicating that recovery was continuing long past the end of the transition care program episode of care.

Summary scores for the EQ-5D and ICECAP-O are presented in Table 2. At all time points the utility values derived from the ICECAP-O were higher than those derived using the EQ-5D. The increase in ICECAP-O over time was significant (p < .01) and non-significant for the EQ-5D (p = .80).

**Table 2:** Utility scores over the six month follow up

	**Initial**	**End of TCP**	**Three months**	**Six months**	**GEE effect × time**	**Total QALYs**	Incremental QALYs*
N	349	317	302	314		346	346
EQ-5D	0.55 (0.20)	0.70 (0.20)	0.60 (0.25)	0.60 (0.28)	0.006 (−.040, 0.053)	0.28 (0.10)	0.0021 (0.0469)
N	343	318	311	312		339	339
ICECAP-O	0.75 (0.16)	0.83 (0.16)	0.83 (0.23)	0.84 (0.25)	**0.027 (0.009, 0.045)**	0.38 (0.08)	0.0087 (0.0315)

Notes: TCP = transition care program; GEE = generalized estimating equation QALY = quality adjusted life year; Bold = significant result.

* calculated as area under the curve adjusted from baseline.

Total QALYs (or time × utility) and incremental QALYs gained for the two instruments for the six month period are listed in Table 2. The incremental QALYs gained showed a small positive gain over the six month period. The instruments yielded different estimates of this gain with the ICECAP-O delivering a significantly higher overall gain (p = 0.02).

The type of services, frequency (defined as percentage of cohort who received the service) and the intensity (measured as hours of care) provided to the cohort are presented in Table 3. The three most frequently provided services (apart from Case Management) were Physiotherapy, Occupational Therapy and Personal Care. The most hours of care given however were services for Personal Care followed by Home Care and Occupational Therapy. The total hours of care provided represent an average of 12 hours per week per person for an average seven week episode of care with about nine and a half being direct patient contact and two and a half comprising Case Management.

**Table 3:** Service characteristics

**Service**	**Percentage of patients who received that service**	Time spent in hours (mean sd)*
Case Management	100%	19.86 (12.44)
Physiotherapy	92%	9.41 (8.76)
Occupational Therapy	88%	11.28 (11.70)
Personal Care	87%	29.04 (29.20)
Nursing	76%	8.88 (11.71)
Home Care	37%	16.92 (19.74)
Allied Health Assistant	37%	7.71 (8.36)
Social Work	35%	4.30 (5.29)
Dietetics	33%	3.74 (3.65)
Podiatry	29%	1.45 (3.13)
Total Direct Contact Time**	100%	66.23 (52.74)

Notes: *Represents the service time in hours per person for the duration of the transition care episode of care (average seven weeks). Expected value can be obtained by multiplying the service time by the percentage of patients who received that service.

**Direct Contact time does not include Case Management time and represents the average per person in the study.

Table 4 presents the results of the regression analysis. Both the EQ-5D and ICECAP-O demonstrated positive associations between the amount of direct contact time provided to the patient and QALYs gained. This yielded remarkably consistent, although slightly different, results between the EQ-5D and ICECAP-O with Personal Care Workers time having the most significant effect on both measures followed by Physiotherapy and Home Care for the EQ-5D and Occupational Therapy and Physiotherapy for the ICECAP-O.

**Table 4:** Results of the regression analyses

	**EQ-5D**		**ICECAP-O**	
**Service time**	**Coefficient (95% CI)**	**P**	**Coefficient (95% CI)**	**P**
Total direct contact time	**0.18 (0.08, 0.27)**	**<0.01**	**0.09 (0.03, 0.15)**	**0.01**
Case management	0.19 (−0.23, 0.6)	0.37	0.16 (−0.12, 0.43)	0.25
Nursing	0.16 (−0.32, 0.62)	0.52	−0.07 (−0.38, 0.25)	0.68
Physiotherapy	**0.93 (0.36, 1.49)**	**<0.01**	**0.42 (0.04, 0.8)**	**0.03**
Occupational Therapy	0.37 (−0.08, 0.8)	0.10	**0.38 (0.09, 0.67)**	**0.01**
Personal Care Worker	**0.34 (0.17, 0.51)**	**<0.01**	**0.17 (0.05, 0.28)**	**0.01**
Home Care Worker	**0.36 (0.01, 0.7)**	**<0.05**	0.15 (−0.09, 0.38)	0.22
Dietician	−1.1 (−2.94, 0.76)	0.25	−1.2 (−2.43, 0.03)	0.06
Podiatry	0.28 (−2.6, 3.15)	0.85	0.5 (−1.42, 2.42)	0.61
Social Work	−0.39 (−1.74, 0.98)	0.58	−0.4 (−1.31, 0.51)	0.39
Allied Health Assistant	0.24 (−0.57, 1.04)	0.56	0.36 (−0.18, 0.89)	0.19

Notes: Service time represents the time in hours received by a patient of each service type. All co-efficients are x10-3 therefore co-efficients represent potential gain in QALYs per 1000 hours of input. Statistically significant results appear in bold.

## Discussion

It is well known that older people are functionally worse and have poor quality of life following extended admissions to hospital [3,20,21]. However, it has not been known how much recovery has been possible. This study shows that older people receiving a post-discharge program continue to recover from acute episodes over time with improvements in QoL spanning at least six months. Death rates were substantially above Australian averages; however, this is not surprising given the frail nature of the participants.

Major improvements were for the mobility, self care and usual activities domains of the EQ-5D and for role and enjoyment dimensions of the ICECAP-O. Interestingly, recovery in these domains continued post the end of the supportive transition care program. Although the EQ-5D and ICECAP-O were developed from different theoretical constructs and the valuation methods are also very different, both instruments yielded consistent results with the direction of health gain although only the ICECAP-O showed significant differences over time. These results could support the developers of the ICECAP-O’s view that health related QoL measures such as the EQ-5D are too narrow to capture benefits for older people and that the ICECAP-O could provide additional useful information to evaluate social and community services [16].

This is the first research to demonstrate the impact of intensity of a slow stream rehabilitation and support program measured in time provided for older people on utility. Previous studies have found some positive influence on QoL of community programs for older frail people. A small study found that twice weekly water exercise maintained QoL better than once weekly measured by the SF-36 in a group of older adults with mobility related Activities of Daily Living dependence [22]. An Australian study examining the effect of a post discharge 24-week program of nursing and physiotherapy found significant improvements in both QoL and re-admission rates over a “do nothing” alternative [23].

This analysis extends previous research by showing that changes in utility are positively related to the amount of input in terms of direct patient care provided. Additionally, the study demonstrates that the specific inputs most related to change are personal care workers, physiotherapy, occupational therapy and home care. These factors should be considered when developing similar programs.

A major limitation is the observational nature of the study. It cannot be determined how much of the improvement observed was due to the influence of the program and how much would be due to natural recovery following hospitalisation. No control group was available for this study. A randomised controlled trial of the transition care program is not possible as it is unethical to potentially deny patients a service that already exists by incorporating a control group into a study of the service. It may be possible that without any input post-acute, older people may be at risk of further deterioration and higher rates of residential care or they may spontaneously recover from these episodes. Therefore the incremental QALY improvement over a “do nothing” alternative may be higher or lower than the incremental QALYs from baseline estimated in this study.

The study was conducted in six different sites, which may have provided slightly different models of care. While this may have impacted on the results, there is variation in program delivery within each site, since the program is tailored to individual patient needs. The cohort was considered representative of TCP recipients, having been recruited across sites in both rural and metropolitan communities.

## Conclusions

The type and intensity of programs offered to older frail people post hospital admission can impact on their recovery and quality of life gains. In particular providing personal care, physiotherapy and occupational therapy appear to offer the best gains. This should be considered when developing similar services for older people.

While the utility gains over time appear small and therefore not clinically relevant, the analysis cannot take into account the likely effect of the transition care program on maintenance of quality of life in people who otherwise may have declined over the six months. Therefore the incremental QALY gain of the program may likely be higher than the estimates obtained from this observational study.

The utility values derived will provide useful baseline information for other researchers on the utility of older people following a prolonged hospital stay and demonstrate the amount of potential recovery available in a six month period for a group of frail older people.

## Abbreviations

TCP: Transition Care Program; QoL: Quality of Life; RAC: Residential Aged Care; EQ-5D: European Quality of life Five Dimensions; SF-36: Short Form Health Survey 36; HREC: Human Research Ethics Committee; ICECAP-O: Investigating choice experiments for the preferences of older people CAPability instrument; UK: United Kingdom.

## Competing interests

The authors certify that no party having a direct interest in the results of the research supporting this article has or will confer a benefit on us or on any organisation with which we are associated. The funding sources had no involvement in the design, execution, analysis and interpretation of data, nor writing of the paper.

## Authors’ contributions

TC responsible for writing article, data analysis and interpretation. NP responsible for study design, implementation, data analysis and editing paper. LG and PS contributed to study design, writing and editing paper. All authors read and approved the final manuscript.

## References

[B1] Stott DJ Langhorne P Knight PV Multidisciplinary care for elderly people in the community Lancet 2008 371 9614 699 700 10.1016/S0140-6736(08)60317-7 18313484

[B2] Borowiak E Kostka T Predictors of quality of life in older people living at home and in institutions Aging-Clinical & Experimental Research 2004 16 3 212 220 15462464 10.1007/BF03327386

[B3] Giles LC Hawthorne G Crotty M Health-related quality of life among hospitalized older people awaiting residential aged care Health And Quality Of Life Outcomes 2009 7 71 71 10.1186/1477-7525-7-71 19630996 PMC2725036

[B4] Gray LC Travers CM Bartlett HP Crotty M Cameron ID Transition care: will it deliver? Med J Aust 2008 188 4 251 253 18279139 10.5694/j.1326-5377.2008.tb01600.x

[B5] Crotty M Whitehead CH Wundke R Giles LC Ben-Tovim D Phillips PA Transitional care facility for elderly people in hospital awaiting a long term care bed: randomised controlled trial BMJ 2005 331 7525 1110 10.1136/bmj.38638.441933.63 16267077 PMC1283272

[B6] Peel NM Hubbard RE Gray LC Impact of post-acute transition care for frail older people: a prospective study Journal of Frailty and Aging 2013 EPub ahead of print 30/1/2013 10.14283/jfa.2013.24 27070816

[B7] Gray LC Berg K Fries BE Henrard JC Hirdes JP Steel K Morris JN Sharing clinical information across care settings: the birth of an integrated assessment system BMC Health Serv Res 2009 9 71 10.1186/1472-6963-9-71 19402891 PMC2685125

[B8] Rockwood K Mitnitski A Frailty in relation to the accumulation of deficits J Gerontol A Biol Sci Med Sci 2007 62 7 722 727 10.1093/gerona/62.7.722 17634318

[B9] Singh I Gallacher J Davis K Johansen A Eeles E Hubbard RE Predictors of adverse outcomes on an acute geriatric rehabilitation ward Age Ageing 2012 41 2 242 246 10.1093/ageing/afr179 22301571

[B10] Davis JC Marra CA Robertson MC Khan KM Najafzadeh M Ashe MC Liu-Ambrose T Economic evaluation of dose-response resistance training in older women: a cost-effectiveness and cost-utility analysis Osteoporos Int 2011 22 5 1355 1366 10.1007/s00198-010-1356-5 20683707 PMC4508130

[B11] Holland R Smith RD Harvey I Swift L Lenaghan E Assessing quality of life in the elderly: a direct comparison of the EQ-5 day and AQoL Health Econ 2004 13 8 793 805 10.1002/hec.858 15322991

[B12] Comans TA Currin ML Brauer SG Haines TP Factors associated with quality of life and caregiver strain amongst frail older adults referred to a community rehabilitation service: implications for service delivery Disabil Rehabil 2011 33 13–14 1215 1221 20977392 10.3109/09638288.2010.525288

[B13] Coast J Peters TJ Natarajan L Sproston K Flynn T An assessment of the construct validity of the descriptive system for the ICECAP capability measure for older people Qual Life Res 2008 17 7 967 976 10.1007/s11136-008-9372-z 18622721

[B14] Coast J Flynn TN Natarajan L Sproston K Lewis J Louviere JJ Peters TJ Valuing the ICECAP capability index for older people Social Science and Medicine 2008 67 5 874 882 10.1016/j.socscimed.2008.05.015 18572295

[B15] Viney R Norman R King MT Cronin P Street DJ Knox S Ratcliffe J Time trade-off derived EQ-5D weights for Australia Value in Health 2011 14 6 928 936 10.1016/j.jval.2011.04.009 21914515

[B16] Coast J Flynn TN Natarajan L Sproston K Lewis J Louviere JJ Peters TJ Valuing the ICECAP capability index for older people Soc Sci Med 2008 67 5 874 882 10.1016/j.socscimed.2008.05.015 18572295

[B17] Twisk JW A comparison between generalized estimating equations and random coefficient analysis. Longitudinal data analysis Eur J Epidemiol 2004 19 8 769 776 15469034 10.1023/b:ejep.0000036572.00663.f2

[B18] Manca A Hawkins N Sculpher MJ Estimating mean QALYs in trial-based cost-effectiveness analysis: the importance of controlling for baseline utility Health Econ 2005 14 5 487 496 10.1002/hec.944 15497198

[B19] Australian Bureau of Statistics 3302.0.55.001 - Life tables; 2007-2009 2010 Canberra: Australian Government http://www.abs.gov.au/ausstats/abs@.nsf/mf/3302.0.55.001

[B20] Lakhan P Jones M Wilson A Courtney M Hirdes J Gray LC A prospective cohort study of geriatric syndromes among older medical patients admitted to acute care hospitals J Am Geriatr Soc 2011 59 11 2001 2008 10.1111/j.1532-5415.2011.03663.x 22092231

[B21] Mudge AM Kasper K Clair A Redfern H Bell JJ Barras MA Dip G Pachana NA Recurrent readmissions in medical patients: a prospective study J Hosp Med 2011 6 2 61 67 10.1002/jhm.811 20945294

[B22] Sato D Kaneda K Wakabayashi H Nomura T Comparison two-year effects of once-weekly and twice-weekly water exercise on health-related quality of life of community-dwelling frail elderly people at a day-service facility Disabil Rehabil 2009 31 2 84 93 10.1080/09638280701817552 18608400

[B23] Courtney M Edwards H Chang A Parker A Finlayson K Hamilton K Fewer emergency readmissions and better quality of life for older adults at risk of hospital readmission: a randomized controlled trial to determine the effectiveness of a 24-week exercise and telephone follow-up program J Am Geriatr Soc 2009 57 3 395 402 10.1111/j.1532-5415.2009.02138.x 19245413

